# Inflammation and Elevated Osteopontin in Plasma and CSF in Cerebral Malaria Compared to *Plasmodium*-Negative Neurological Infections

**DOI:** 10.3390/ijms25179620

**Published:** 2024-09-05

**Authors:** Monique F. Stins, Agnes Mtaja, Evans Mulendele, Daniel Mwimbe, Gabriel D. Pinilla-Monsalve, Mable Mutengo, Carlos A. Pardo, James Chipeta

**Affiliations:** 1 Malaria Research Institute, Johns Hopkins School of Public Health, 615N Wolfe Street, Baltimore, MD 21205, USA; 2 Biomedical Research Institute of Southern California, Oceanside, CA 92046, USA; 3 University Teaching Hospital Malaria Research Unit (SMUTH-MRU), Department of Pediatrics and Child Health, University of Zambia School of Medicine, Lusaka P.O. Box 50110, Zambia; 4 Division of Neuroimmunology and Neuroinfectious Diseases, Department of Neurology, Johns Hopkins School of Medicine, 600 N Wolfe Street, Baltimore, MD 21285, USA; gpinill1@jhmi.edu (G.D.P.-M.); cpardov1@jhmi.edu (C.A.P.); 5 Department of Radiology, Faculty of Medicine, University of Montreal, 2900 Edouard Montpetit Blvd, Montreal, QC H3T 1J4, Canada; 6 Institute of Basic and Biomedical Sciences, Levy Mwanawasa Medical University, Lusaka P.O. Box 33991, Zambia

**Keywords:** cerebral malaria, meningitis, inflammation, osteopontin, IP10, GRO, brain

## Abstract

Cerebral malaria in young African children is associated with high mortality, and persisting neurological deficits often remain in survivors. Sequestered *Plasmodium*-infected red blood cells lead to cerebrovascular inflammation and subsequent neuroinflammation. Brain inflammation can play a role in the pathogenesis of neurologic sequelae. Therefore, we assessed a select set of proinflammatory analytes (IP10, IL23, MIP3α, GRO, MCP-1, and osteopontin in both the plasma and cerebrospinal fluid(CSF) of Zambian children with cerebral malaria and compared this with children with neurological symptoms that were negative for *Plasmodium falciparum* (non-cerebral malaria). Several similarities in plasma and CSF levels were found, as were some striking differences. We confirmed that IP10 levels were higher in the plasma of cerebral malaria patients, but this was not found in CSF. Levels of osteopontin were elevated in both the plasma and CSF of CM patients compared to the non-CM patients. These results show again a highly inflammatory environment in both groups but a different profile for CM when compared to non-cerebral malaria. Osteopontin may play an important role in neurological inflammation in CM and the resulting sequelae. Therefore, osteopontin could be a valid target for further biomarker research and potentially for therapeutic interventions in neuroinflammatory infections.

## 1. Introduction

Cerebral malaria (CM) is a clinical syndrome caused by the *Plasmodium falciparum* infection and is associated with a high mortality rate (particularly in children). Those who survive frequently suffer from neurological sequelae, including epilepsy and cognitive disabilities [1,2,3]. CM is linked to a heightened inflammatory state, along with the adherence of *Plasmodium falciparum*-infected red blood cells (PRBCs) to the walls of cerebral blood vessels, leading to the blockage of some vessels. Interestingly, PRBCs do not cross the blood–brain barrier (BBB) into the brain. The sequestration of PRBCs in the cerebral vessels causes brain endothelial inflammation, thus contributing to an altered balance of growth factors and pro-inflammatory analytes released into the brain [4]. While inflammatory mediators in the blood of CM patients have been documented, data are scarce on their levels in cerebrospinal fluid (CSF). Therefore, this study aimed to assess multiple inflammatory mediators in both the blood and CSF of CM patients and to compare these with patients with neurological symptoms but without *Plasmodium* infection. Specifically, we assessed the levels of interferon-inducible protein of 10 Kda (IP10), interleukin 23 (IL23), macrophage inducible protein 3α (MIP3α), macrophage chemotactic protein-1 (MCP-1), growth-related oncogene (GRO) chemokines, and osteopontin (OPN).

IP10 or CXCL10 is a chemokine induced by TNFα and IFNγ and has been implicated in various neuroinflammatory conditions, including viral encephalitis [5,6], bacterial meningitis [7], and CM [8,9,10], where it serves as a potential biomarker for mortality prediction [11]. OPN is a broadly expressed pleiotropic glycoprotein with a glycine–arginine–glycine–aspartate–serine (GRGDS) cell-binding domain and is present in various tissues, including endothelial cells, and can be found in urine and breast milk [12,13,14,15,16]. OPN has important roles in tissue homeostasis, cellular proliferation and differentiation, stress responses, and the regulation of both systemic and central nervous system (CNS) innate and adaptive immune responses [17]. OPN has been shown to be involved in several brain infections [13,18,19,20]. Animal studies showed that the role of OPN can be protective, as increased mortality for malaria was observed in OPN knock-out mice [20]; moreover, OPN has been known to reduce the entry of West Nile virus into the CNS [21]. However, to date, there is no information on OPN levels in CM.

GRO chemokines (GROα, GROβ, GROγ or CXCL1, CXCL2, CXCL3) increase during inflammation both in blood and CSF. Astrocytes have been shown to secrete GROα and its receptor (CXCR2) is present in neurons [22,23]. Several neuronal effects of GRO chemokines have been previously shown, such as the regulation of the functional properties of AMPA-type glutamate receptors through CXCR2, which results in the increase in affinity of glutamate receptors for the neurotransmitter and the amplitude of postsynaptic transmission [24]. In addition, GRO chemokines modulate Purkinje neuron activity [25] and increase ERK phosphorylation in cortical neurons [22]. However, the GRO chemokine levels in CM are unknown, as well as the role they may play in CM.

Understanding changes in chemokine and OPN levels could substantially contribute to elucidating the mechanisms underlying neurological dysfunction and sequelae in CM. In this study, we collected plasma and CSF samples from children with CM to explore specific responses attributable to *Plasmodium* infection, comparing these profiles with those found in non-*Plasmodium*-infected patients presenting with neurological deficits.

## 2. Results and Discussion

Socio-demographic, clinical, and laboratory variables are described in Table 1 and Table 2, as previously reported [26]. Briefly, participants of this study had an age of 4 years (interquartile range, IQR 2.31–6.10), weighed 14 kg (IQR 10.80–25.10), and exhibited a body temperature of 38.8 °C (IQR 37.70–39.20). There were no significant clinical differences observed between the CM and non-CM groups, except for the frequency of pallor (63.64% vs. 11.76%; *p* = 0.010) and the scores in the pediatric Glasgow Coma Scale (GCS) (8; IQR 8–9 vs. 10; IQR 9–15 points; *p* = 0.015).

Differences were noted in the RBC volume (3.51 × 10^6^ RBC/µL; IQR 3.23–4.25 in the CM group versus 4.42 × 10^6^ RBC/µL; IQR 4.05–4.67 in the non-CM group; *p* = 0.034). Thrombocytopenia was identified in the CM group with 67.5 × 10^9^/µL (IQR 59–161) for CM and 246 × 10^9^/µL (IQR 193–383) for the non-CM patient group (*p* = 0.009). In addition, a higher percentage of the non-CM patients demonstrated an abnormal WBC count in CSF (n = 9, 52.49%) than the CM patients (n = 1, 9.09%) (*p* = 0.041).

This study demonstrates a profound inflammatory response in both CM and non-CM neurological infections, evidently present in both blood and CSF. Overall, elevated levels of inflammatory cytokines and chemokines were observed across analytes in both patient groups, yet notable distinctions were identified in IP10 and OPN levels (plasma and CSF concentrations are available in Supplementary Table S1).

### 2.1. Analyte Differences between the CM and Non-CM Group

#### 2.1.1. Higher IP10 Levels in Plasma in CM but Not in CSF

**Figure 1:**
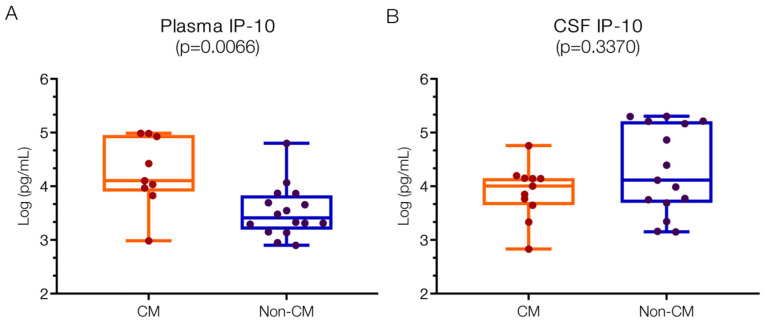
**Higher IP10 values in the plasma but not in the CSF of children with CM versus non-CM**. (**A**) Levels of IP10 in the plasma of CM patients are significantly higher than those in non-CM patients (*p* = 0.0066). (**B**) Levels of IP10 in CSF of CM and non-CM patients do not differ significantly (*p* = 0.3370). Log10 values are indicated on the *y*-axis.

To discern variations between CM and non-CM neurological infections, IP10 levels were assessed in plasma and CSF (Figure 1). Plasma IP10 levels were significantly higher in CM patients (4.11; IQR 3.97–4.93 log pg/mL) compared to the non-CM group (3.41; IQR 3.23–3.79 log pg/mL; *p* = 0.007). These findings align with previous reports indicating elevated IP10 in CM patients, surpassing levels reported by Armah et al. [8].

In contrast, CSF IP10 levels did not mirror the plasma findings, showing numerically higher levels in the non-CM group. Specifically, CM patients exhibited IP10 levels in the CSF of 4.01 (IQR 3.65–4.15) log pg/mL, whereas non-CM patients displayed levels of 4.12 (IQR 3.69–5.21) log pg/mL. Previous studies have reported IP10 elevations of approximately 1.4 (1–2) log pg/mL in the CSF of CM patients [11], contrasting sharply with negligible levels in non-malarial controls, underscoring the significance of our findings.

The discrepancy between IP10 levels in plasma versus CSF suggests differential cellular sources of IP10 in the brain and periphery among these patient groups. For instance, both astrocytes and microglia are implicated in IP10 release, with astrocytes responding to viral infections like HIV and Japanese encephalitis virus, while microglia release IP10 during bacterial infections, LPS exposure, and CMV infections [27,28,29].

Moreover, the involvement of IP10 also indicates a pathogenic role for activated T cells in the CNS [30]. Experimental murine models for CM have implicated T cell activity in CNS pathology [31], and this has been shown in human CM as well [32]. Variations in plasma/CSF IP10 balance also reflect differences in circulating versus infiltrating immune cells, potentially influenced by abnormal white blood cell (WBC) counts observed more frequently in the CSF of non-CM patients (52.94% versus 9.09%).

#### 2.1.2. Elevated GRO Chemokines in Both CM and Non-CM Patients

**Figure 2:**
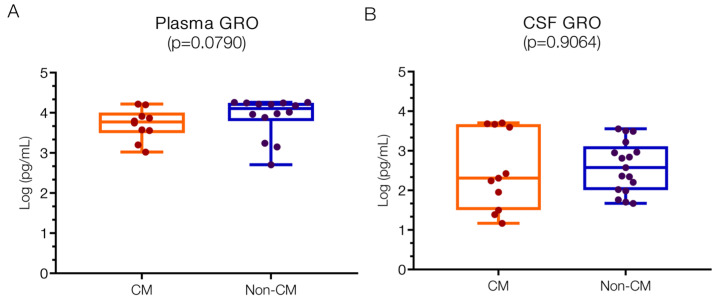
**Similar plasma and CSF values of GRO chemokines in children with CM versus non-CM**. GRO values are not significantly different in the plasma (**A**) or in the CSF (**B**) of patient groups, although there is a greater variation in the CSF of non-CM patients. Log10 values are indicated on the *y*-axis.

There were minor numeric differences in GRO levels that did not reach statistical significance when comparing the two patient groups. The median levels of GRO chemokines were marginally lower in the CM group for both plasma and CSF. The levels of GRO chemokines in plasma (Figure 2A) were 3.77 (IQR 3.56–3.91) log pg/mL for CM and 4.11 (IQR 3.89–4.25) log pg/mL for non-CM patients, above reported normal physiological levels (approximately 1.2 log pg/mL) but also higher than in *Plasmodium*-infected patients from Saudi Arabia (approximately 1; 0.3–1.2 log pg/mL) [33].

In CSF (Figure 2B), GRO levels were generally lower compared to plasma, with a wider range observed in the CM group (2.31; IQR 1.50–3.67 log pg/mL) versus the non-CM group (2.58; IQR 2.02–2.97 log pg/mL). At present, no comparative data on GRO levels in CSF from healthy individuals could be located.

While human malaria studies lack specific reports on GRO chemokine levels, increased levels have been documented in bacterial meningitis [7,33] and in viral encephalopathies [34]. Animal studies indicate elevated GROα and GROβ levels in infections with Theiler virus [35], JC virus [36], and Rift Valley virus [37], suggesting heterogeneous cellular sources in the CNS depending on the analyzed brain region and species. Ischemic injury, for instance, elevates GROα in capillary endothelial cells and CD68+ microglia, but not in astroglia [38].

Studies also point to amoeboid microglia and oligodendrocyte precursor cells (OPCs) as potential sources in humans [23], with brain endothelial cells induced by Bacillus anthracis and PRBCs in in vitro BBB models [39,40,41]. Astrocytes were found to respond to *E. coli* by releasing GROα [42], whereas others found that a bacterial challenge also favors the release of GRO chemokines from both a subset of neurons and astrocytes but not from microglia [43]. Other animal studies also point to astrocytes as a source of GROα [44,45]. Which type of CNS cells are involved in GRO chemokine release in CM versus non-CM is not clear and would need further investigation.

The specific cellular contributors to GRO chemokine release in CM versus non-CM/meningitis remain unclear and warrant further investigation. CXCR2, the receptor for GRO, is broadly expressed on cortical neurons axons (including those in the hippocampus) [46,47], and a subset of astrocytes [48], suggesting functional and communicative interactions between GRO-producing cells and receptor-bearing neurons. Moreover, GRO chemokines influence neurotransmitter release [49] and enhance synaptic currents in neuronal cultures [49], implicating their potential role in the development of neurological symptoms in CM and non-CM conditions.

#### 2.1.3. Detected Levels of MIP3-α, IL23, and MCP1 in Both CM and Non-CM Patients

**Figure 3:**
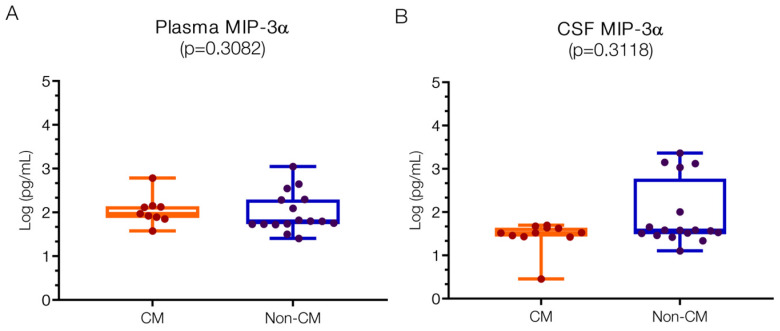
**Similar MIP-3α levels in plasma and CSF in children with CM versus non-CM**. (**A**) No statistical differences were found in the MIP-3α plasma values. (**B**) Although the values of MIP-3α in the CSF show a greater spread in the non-CM patients than in the CM patients, there is no statistical difference. Log10 values are indicated on the *y*-axis.

Plasma levels of MIP-3α were 1.97 (IQR 1.89–2.13) log pg/mL in the CM group and 1.8 (1.73–2.29) log pg/mL in the non-CM group, with no significant difference observed between them (Figure 3A). These levels align closely with reported normal physiological ranges (1.95 log pg/mL), and similar concentrations have been noted in severe malaria [50] and tick-borne meningitis of around 2.25 ± 2.52 pg/mL [51]. While there was greater variability in the CSF-MIP-3α levels in the non-CM group (1.58; IQR 1.51–2.00 log pg/mL) compared to the CM group (1.52; IQR 1.44–1.64 log pg/mL) (Figure 3B), this difference was not statistically significant. The heterogeneity in CSF-MIP-3α levels within the non-CM group, encompassing conditions such as acute bacterial meningitis versus non-etiological encephalopathy, likely contributes to this variation.

**Figure 4:**
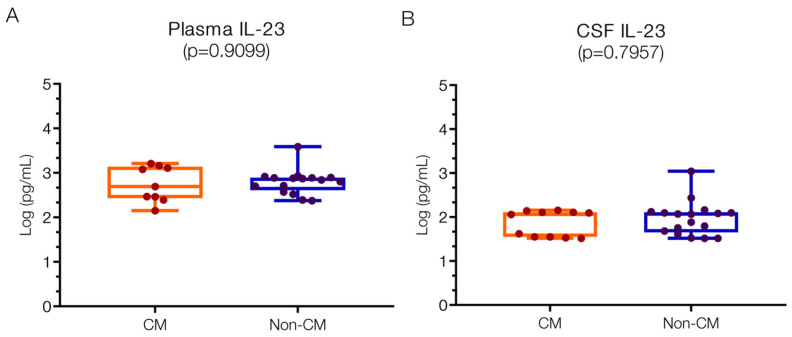
**Similar IL23 levels in plasma and CSF in children with CM versus non-CM**. IL23 levels both in plasma (**A**) and CSF (**B**) are not significantly different in CM and non-CM. Log10 values are indicated on the *y*-axis.

Plasma IL23 levels were 2.69 (IQR 2.47–3.11) log pg/mL in the CM group and 2.86 (IQR 2.64–2.89) log pg/mL in the non-CM group (Figure 4A), with no significant difference noted between the groups. These levels exceed reported normal ranges (0–1.8 log pg/mL) [7], suggesting their involvement in human CM pathogenesis through mechanisms potentially related to reduced nitric oxide (NO) production [52,53]. While animal studies indicate no direct role in CM development and mortality [54], IL23 may still contribute to the formation of neurological sequelae. In the CSF, IL23 levels did not differ significantly between the CM (2.06; IQR 1.55–2.11 log pg/mL) and non-CM groups (2.07; IQR 1.69–2.10 log pg/mL) (Figure 4B). In contrast, negligible levels are reported in non-infected controls, while substantial increases have been observed in bacterial meningitis (up to 1.94; 1.5–2.44 log pg/mL), consistent with our findings.

**Figure 5:**
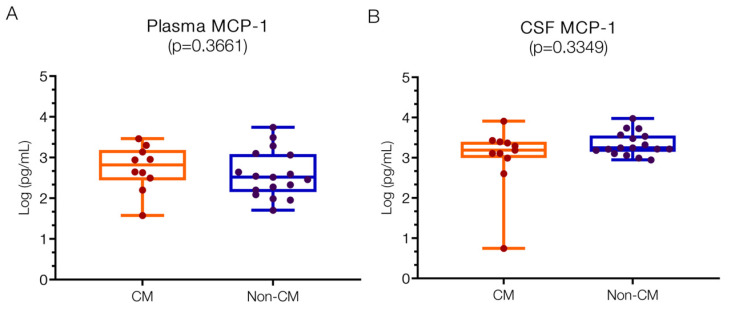
**Similar MCP1 levels in CSF and plasma in children with CM versus non-CM**. MCP1 levels in plasma (**A**) and CSF (**B**) of CM and non-CM patients do not show statistical differences. Log10 values are indicated on the *y*-axis.

The plasma levels for MCP1 were 2.82 (IQR 2.50–3.14) log pg/mL in the CM group and 2.52 (IQR 2.20–3.06) log pg/mL in the non-CM group (Figure 5A), with no significant difference found between them. These levels are within the reported normal range (2; 1.9–2.1 log pg/mL) [11], and studies have not shown important increases in postmortem CM patients compared to controls. The CSF levels of MCP1 levels were 3.19 (IQR 2.99–3.40 log pg/mL) in the CM group and 3.25 (IQR 3.18–3.53) log pg/mL in the non-CM group (Figure 5B), with no significant difference observed between the groups. In bacterial meningitis, base levels are low or near zero [7], and while a study reported levels in CM (0.3 log pg/mL) [11], these were not significantly different in comparison to controls.

In the CNS, MCP-1, together with MIP1α and the GRO chemokines, plays a role in the proliferation, migration, and differentiation of neural progenitors [23,45,55,56,57,58,59,60]. Given the neuronal damage observed in both CM and non-CM meningitis alongside elevated chemokine levels, it is plausible that these factors contribute to neuronal repair and glial differentiation [60,61].

#### 2.1.4. Elevated OPN Levels in Both Plasma and CSF of CM Patients

**Figure 6:**
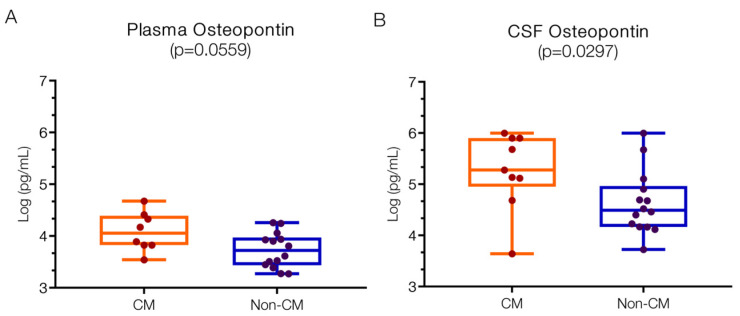
**OPN levels in both plasma and CSF are higher in children with CM versus non-CM**. (**A**) Numerically elevated levels of peripheral OPN in the plasma of CM patients (*p* = 0.0559). (**B**) Particularly, the CSF levels of OPN are significantly increased in the CM compared to non-CM (*p* = 0.0297). Log 10 values are indicated on the *y*-axis.

Figure 6A presents the OPN levels in both CM and non-CM groups. The plasma level of OPN in CM patients was 4.05 (IQR 3.83–4.37) log pg/mL, compared to 3.72 (IQR 3.45–3.94) log pg/mL in non-CM patients. Burdo et al. [62] found higher plasma OPN levels in HIV patients (2.8; 2.6–3 log pg/mL) compared to control plasma (2.55; 2.63–3.45 log pg/mL). Hayek et al. [63]. reported baseline plasma OPN levels of 4.22 log pg/mL in control patients, which increased to 4.99 log pg/mL in those with SARS-CoV infections (COVID-19). In Uganda, plasma OPN levels in pregnant mothers and babies from *Plasmodium*-endemic areas ranged between 4–5 log ng/mL [64]. These published baseline OPN values vary considerably due to factors such as sample type (plasma/serum), anticoagulant type, assay type, antibodies used, OPN fragment detected, and population geographic location [64].

Figure 6B shows OPN levels in the CSF, with 5.28 (IQR 5.12–5.90) log pg/mL in the CM group and 4.49 (IQR 4.17–4.91) log pg/mL in the non-CM group, which is significantly different (*p* = 0.0297). Burdo et al. [62]. reported control CSF levels of 3.2 (2.95–2.45) log pg/mL, increasing to 3.6 (3.35–3.75) log pg/mL in patients suffering from HIV infection with and without associated dementia [65]. A study of sleeping sickness patients and parasite-negative controls found higher CSF values of 5.1 (4.78–5.4) log pg/mL, which increased to 5.9 (5.8–6.27) log pg/mL [65] in infected subjects. Although different control values exist, OPN levels are consistently increased in the CSF of infected patients.

In addition to elevated IP10, cytokine, and chemokine levels, high OPN levels were observed in both CM and non-CM patients. In both the plasma and CSF of the CM group, OPN levels were numerically higher than in the non-CM group, but the CSF to plasma ratios of OPN were comparable (1.26 IQR 0.51–2.08 versus 0.75 IQR 0.46–1.75, respectively, *p* = 0.221). Currently, there is no information on OPN levels in plasma or CSF in human CM or its effects. However, OPN levels are increased in other infections, including bacterial sepsis, COVID-19, and HIV, in both plasma [62,66] and CSF [62,65].

Overall, OPN concentrations appear to be associated with the stage of infection, with higher levels linked to worse outcomes and neuropsychological symptoms, as demonstrated in Trypanosome-infected patients [19,65]. In cases with a higher Trypanosome burden, sharp increases in CSF-OPN levels were observed, even though plasma OPN levels did not change, suggesting a compartmentalized response for OPN. As OPN levels in CSF increased further, more severe neurological symptoms emerged [19]. Interestingly, in HIV-infected patients, plasma OPN correlated with the neuropsychological status of the patients, but this was not the case for CSF levels. Similarly, increased plasma OPN levels positively correlated with neuropsychological scores in SIV [62] and predicted worse outcomes in sepsis and COVID-19 infections [63,67].

Depending on the type of infection, organ, and cells involved, OPN can exert its effects in several ways and have pro- or anti-inflammatory properties [17,21]. Additionally, OPN can increase endothelial permeability, resulting in tissue edema [68,69]. However, some studies showed a protective effect of OPN on BBB integrity after subarachnoid hemorrhage [70,71]. In experimental CM studies, OPN potentially plays a protective role, as KO-OPN models showed increased mortality from *P. chaubaudi* [64]. Whether OPN has a protective effect on the BBB in CM versus non-CM needs further elucidation.

In the CNS, OPN is constitutively expressed at low levels in neurons, microglia, astrocytes, and oligodendrocytes, but it is upregulated and secreted during neuroinflammatory responses [12,17,72,73,74]. Once secreted, OPN can serve as a chemotactic agent for a variety of cell types and signals through integrins and CD44 [17]. OPN can be cleaved by either metalloproteases or caspases, exposing different OPN domains (see review by Capellano et al. [75]). The resulting fragments have a variety of consequences, sometimes with opposing effects, including the differentiation of neural stem cells [76,77] and other neuroprotective mechanisms [78]. Thus, the heterogeneous effects of OPN and the created fragments depend on the local cellular environments and locally active proteolytic enzymes.

If the increase in OPN in CM and non-CM is anti-inflammatory and neuroprotective, OPN supplementation may be beneficial. Several animal studies have shown that milk OPN can be taken up into the brain and is beneficial for brain development by increasing the proliferation and differentiation of oligodendrocyte progenitors [16]. OPN can also be supplemented through the diet (e.g., bovine milk or human breast milk), with the latter containing higher levels of OPN (18 mg/L versus 138 mg/L, respectively). This supplementation could also be advantageous for nursing children. However, if OPN is not neuroprotective, its expression can be pharmacologically downregulated. For example, IFNβ or glatiramer (an immunomodulator) treatment reduced serum OPN levels in multiple sclerosis and ameliorated the course of the disease [79]. Reduced OPN levels were also achieved by atorvastatin (a cholesterol-lowering drug) in diabetic rats [80] and through the suppression of GSK3β activity.

In summary, both CM and non-CM patients exhibit considerable inflammation, with some notable differences. In CM patients, higher levels of OPN in the CSF and increased IP10 levels in plasma were identified. This suggests specific infection-dependent and CNS host-dependent responses that are a combination of excessive inflammatory anti-microbial responses and neuroprotective responses. The increase in levels of IP10, together with the previously reported increase in caspases, may be related to neuronal death, oligodendrocyte apoptosis, and BBB dysfunction [81,82,83,84,85,86]. The levels of the GRO chemokines, along with MCP1 and MIP1α, are likely a neuroprotective response aimed at repair. However, there is an overlapping localization of CXCR2 and CXCR3, so both GRO chemokines and IP10 may modulate each other’s signals.

Although the presence of OPN in CSF may not be disease-specific, further investigation into the role of OPN in CM may reveal potential targets for therapeutic intervention. Due to the existence of three isoforms, future inquiries are needed to determine which isoform is detected in blood versus CSF and at what ratios. This study was conducted with a limited number of participants, and a larger cohort is needed to determine if OPN could be a biomarker and indicator of neurological damage and predictive of post-CM neurological sequelae, either by itself or in combination with other markers. Additionally, since CSF is not easily available, OPN levels in plasma or urine should be further assessed to see if these correlate with post-CM neurological sequelae. Care should be taken to target detrimental inflammation while sparing or encouraging neuroprotective inflammation. Thus, more in-depth research is needed to relate the role of these analytes in neuro-infectious diseases and how they correlate with better post-infection outcomes.

As a result of the exposure to *Plasmodium*-infected red blood cells in CM in comparison to bacteria, viruses, and their toxic products in non-CM, the impairment of endothelial cells leads to a differential release of inflammatory compounds, both into the systemic circulation and the brain. Products released into the brain lead to brain inflammation and neuronal damage that can result in neurologic sequelae.

## 3. Materials and Methods

### 3.1. Participants and Sample Collection

Participants for this study were recruited from the pediatric ward of the University Teaching Hospital, Lusaka, Zambia, as described previously [26]. Children aged between 6 months to 15 years presenting at the hospital with CM symptoms were eligible for enrolment in the study. Participants who qualified as neuroinfectious controls (non-CM diagnosis) had diverse diagnoses, including non-etiological encephalopathy and bacterial meningitis, and were negative for *Plasmodium* infection. Patients who tested positive for HIV during screening or with macroscopic xanthochromia (>1 RBC) were excluded. A total of 12 patients with CM were admitted while 24 patients were included as neurological controls. After the exclusion criteria, 11 CM and 17 non-CM patients remained. As it was not ethical to draw CSF and plasma from healthy children, we were not able to naturalistically assess base levels of analytes.

Children were first stabilized before their parents or caretakers were informed by the research assistant/investigator about the study. From the consenting participants, blood and CSF samples were collected and information concerning demographic and clinical characteristics were recorded. Furthermore, the pediatric Glasgow Coma Scale (GCS) was assessed for each patient. Briefly, this instrument estimates coma/consciousness and motor, verbal, and eye movement scores in 3 categories in a range from 3 to 15. The demographics of the cohort and clinical-paraclinical characteristics are listed in Table 1 and Table 2, as also previously documented [26].

Blood samples (EDTA vacumax, Becton Dickinson, Johannesburg, Gauteng) were collected by venipuncture for cell count, biochemical analyses, and detection of *Plasmodium*, among others. Blood was also collected into blood culture bottles (Bactech, BD Johannesburg, Gauteng) to rule out bacteremia. The remaining blood samples were centrifuged at 1500× *g*, cryopreserved in aliquots of plasma, and stored at −80 °C for cytokine/chemokine analysis using the Luminex Magpix system (Millipore Corp, Burlington, MA, USA). Lumbar puncture was conducted at the L2–L3 or L3–L4 intervertebral space to collect up to 2 mL of sterile cerebrospinal fluid (CSF), which was then stored in polypropylene tubes and transported to the laboratory for standard diagnostic procedures, including bacterial cultures to rule out meningitis. The remaining sample was centrifuged to remove cells, and the supernatant was aliquoted and stored at −80 °C for cytokine/chemokine determination.

### 3.2. Determination of Plasma and CSF Analytes

Analytes were determined using the MagPix (Millipore Corp, Burlington MA, USA) multiplex assays following the manufacturer’s protocol. The kit HCYTOMAG-60K was used for the indicated cytokines and chemokines, while osteopontin was measured using the HND3MAG-39K kit. The assays were performed on a Luminex MAGPIX^®^ instrument with xPONENT^®^ 4.2 MAGPIX^®^ analyzer software.

### 3.3. Statistical Analysis and Graphing

**Figure 7:**
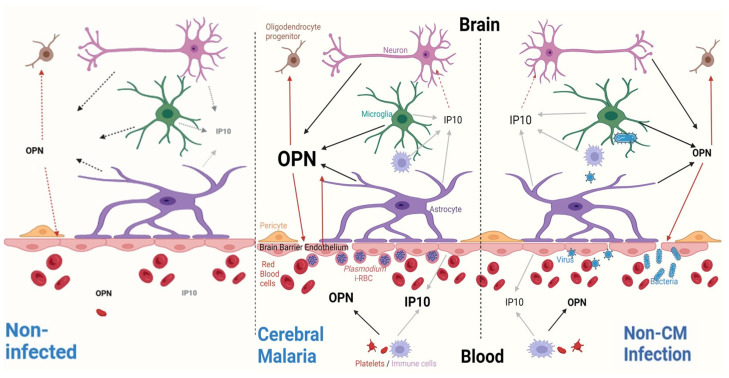
Differential OPN and IP10 responses in CM versus non-CM. Blood (bottom) and brain (top) compartments are separated by endothelial cells of the blood–brain barrier (BBB). Associated pericytes and astrocytes with end feet are in close contact with brain endothelial cells. Cytokines released from endothelial cells and activated microglia lead to high levels of IP10 in the plasma of CM patients and greater variation in the CSF of non-CM patients. GRO chemokines, IL23, MIP-3α, and MCP1 are not statistically different in either condition (not depicted here). However, OPN is numerically higher in the plasma of CM patients and significantly increased in the CSF of the same group.

Data are presented as medians with interquartile ranges (IQR), reflecting the distribution of observations. Comparisons of plasma and CSF non-transformed concentrations were evaluated using Mann–Whitney’s U test, with statistical significance set at *p* < 0.05. Categorical variables were assessed using χ2 or Fisher’s exact test, depending on cell frequencies in the contingency table. Statistical analyses were carried out on Stata 14 (Stata Corp., College Station, TX, USA). Data were log-transformed (Log10) and graphed accordingly. These results were compared with published information about controls (transformed to Log10 values). Graphs were generated using GraphPad Prism, version 7.02 (GraphPad Software, La Jolla, CA, USA), and the graphical abstract (Figure 7) was created using Biorender (biorender.com).

## Figures and Tables

**Table 1:** Demographic and clinical characteristics of the cohort *.

Variable	CM (n = 11)	non-CM (n = 17)	Total (N = 28)	*p* Value
Demographics				
Female (%)	6 (54.55)	6 (35.29)	12 (42.86)	0.444
Age (years)	4.00 (3.11–4.9)	2.50 (2.00–9.00)	4.00 (2.31–6.10)	0.689
Medical history				
Epilepsy (%)	1 (9.09)	0 (0.00)	1 (3.57)	0.393
Cerebral malaria (%)	1 (9.09)	0 (0.00)	1 (3.57)	0.393
Anemia (%)	3 (27.27)	2 (11.76)	5 (17.86)	0.353
Transfusions (%)	0 (0.00)	1 (5.88)	1 (3.57)	>0.999
Symptoms				
Fever (>7 days) (%)	1 (9.09)	2 (11.76)	3 (10.71)	>0.999
Headache (%)	7 (63.64)	11 (64.70)	18 (64.29)	>0.999
Vomit (%)	7 (63.64)	6 (35.29)	13 (46.43)	0.347
Diarrhea (%)	4 (36.36)	7 (41.17)	11 (39.29)	0.703
Cough (%)	3 (27.27)	9 (52.94)	12 (42.86)	0.378
Seizures (%)	8 (72.72)	7 (43.75)	15 (53.57)	0.134
Physical examination				
Weight (kg)	16.00 (13.00–20.00)	12.00 (8.60–26.50)	14.00 (10.80–25.10)	0.384
Temperature (°C)	38.10 (36.90–39.20)	38.90 (37.80–39.20)	38.80 (37.70–39.20)	0.322
Pyrexia (%)	10 (90.91)	16 (94.12)	26 (92.86)	>0.999
Pallor (%)	7 (63.64)	2 (11.76)	9 (32.14)	0.010
Jaundice (%)	3 (27.27)	1 (5.88)	4 (14.29)	0.269
Respiratory distress (%)	1 (9.09)	1 (5.88)	2 (7.14)	>0.999
Pediatric GCS (score)	8.00 (8.00–9.00)	10.00 (9.00–15.00)	9.00 (8.00–12.00)	0.015

* See also Stins et al. [26].

**Table 2:** Laboratory findings for the cohort *.

Variable	CM (n = 11)	non-CM (n = 17)	Total (N = 28)	*p* Value
Blood				
Hemoglobin (g/dL)	8.50 (8.40–9.60)	10.90 (9.20–11.40)	10.20 (8.40–11.40)	0.083
Hematocrit (%)	27.00 (25.10–30.00)	32.70 (29.30–35.50)	31.10 (25.90–35.50)	0.053
Red blood cells (10^6^/μL)	3.51 (3.23–4.25)	4.42 (4.05–4.67)	4.16 (3.50–4.60)	0.034
MCV (fL)	76.25 (74.10–78.60)	76.30 (72.40–83.60)	76.30 (72.40–83.60)	0.802
MCH (pg/cell)	25.00 (24.60–26.60)	26.10 (23.00–26.70)	25.20 (23.30–26.70)	0.802
MCHC (g/dL)	33.05 (32.00–33.90)	32.90 (31.60–34.10)	32.90 (31.60–33.90)	0.919
Platelets (10^9^/L)	67.50 (59.00–161.00)	246.00 (193.00–383.00)	221.00 (70.00–348.00)	0.009
PDW (%)	13.90 (10.90–15.85)	12.45 (11.90–13.65)	12.65 (11.30–14.05)	0.426
White blood cells (10^9^/L)	9.37 (8.80–12.42)	10.97 (10.63–17.41)	10.96 (8.80–13.82)	0.292
Neutrophils (10^9^/L)	5.96 (4.23–7.31)	6.45 (4.52–10.09)	6.04 (4.32–8.26)	0.457
Lymphocytes (10^9^/L)	2.32 (1.84–2.82)	1.67 (1.20–2.67)	2.17 (1.43–2.67)	0.244
Monocytes (10^9^/L)	0.87 (0.76–1.14)	0.69 (0.54–1.34)	0.86 (0.62–1.14)	0.978
Eosinophils (10^9^/L)	0.21 (0.02–0.41)	0.22 (0.02–0.39)	0.22 (0.02–0.41)	>0.999
Basophils (10^9^/L)	0.07 (0.03–0.16)	0.01 (0.01–0.05)	0.04 (0.01–0.06)	0.035
Total protein (g/L)	65.00 (61.00–68.20)	78.10 (71.60–81.30)	70.80 (65.00–79.10)	<0.001
Albumin (g/L)	40.80 (35.40–43.70)	41.60 (33.50–45.80)	41.40 (35.40–45.10)	0.452
ALT (U/L)	39.00 (23.00–46.70)	23.80 (11.00–32.20)	25.40 (18.90–39.20)	0.076
AST (U/L)	72.70 (57.10–142.80)	60.40 (34.10–72.10)	64.25 (36.70–92.30)	0.192
Sodium (mEq/L)	134.00 (132.00–136.00)	132.00 (128.50–135.00)	133.00 (130.00–135.00)	0.330
Chloride (mEq/L)	100.00 (99.00–101.00)	98.20 (96.15–100.95)	99.10 (96.60–101.00)	0.229
Potassium (mEq/L)	4.50 (4.20–5.44)	4.14 (3.58–5.55)	4.20 (3.71–5.44)	0.139
Urea (mmol/L)	5.04 (4.88–8.91)	3.52 (2.76–5.12)	4.59 (3.14–6.44)	0.022
Creatinine (μmol/L)	34.80 (24.50–48.00)	38.90 (31.70–48.90)	38.90 (26.10–48.00)	0.506
Cerebrospinal fluid				
Increased WBC (%)	1 (9.09)	9 (52.94)	10 (35.71)	0.041
Increased RBC (%)	1 (9.09)	7 (41.18)	8 (28.57)	0.099
Glucose (mg/dL)	90.00 (82.62–93.60)	77.49 (71.28–90.36)	88.02 (74.16–91.08)	0.027
Chloride (mEq/L)	123.00 (120.00–126.00)	120.00 (118.00–122.00)	121.00 (119.00–125.00)	0.088

* See also Stins et al. [26].

## Data Availability

Data will be available at request.

## Supplementary Materials

The following supporting information can be downloaded at: https://www.mdpi.com/article/10.3390/ijms25179620/s1.

## Abbreviations

ALT	Alanine transaminase
Ang	Angiopoietin
AST	Aspartate transaminase
BBB	Blood–brain barrier
CM	Cerebral malaria
CNS	Central nervous system
CSF	Cerebrospinal fluid
GCS	Glasgow Coma Scale
GRO	Growth-related oncogene
IFNγ	Interferon gamma
IL	Interleukin
IP10	Interferon-inducible protein of 10kDa
IQR	Interquartile ranges
KDa	Kilo Dalton
L	Lumbar
MCH	Mean corpuscular hemoglobin
MCHC	Mean corpuscular hemoglobin concentration
MCP-1	Macrophage chemotactic proteins-1
MCV	Mean corpuscular volume
MIP1*α*	Macrophage inducible protein 3-alpha
OPN	Osteopontin
PDW	Platelet distribution width
PRBC	*Plasmodium*-infected red blood cells
Q1-Q3	First and third quartiles of the data set.
RBC	Red blood cells
s-ICAM	Soluble intercellular adhesion molecule 1
WBC	White blood cells
