# Supplementary material for: Inflammation and Elevated Osteopontin in Plasma and CSF in Cerebral Malaria Compared to Plasmodium-Negative Neurological Infections
Source: Int J Mol Sci. 2024 Sep 5;25(17):9620. doi: 10.3390/ijms25179620 (PMC11394774; doi:10.3390/ijms25179620)
Supplement: Supplementary file 1 [file ijms-25-09620-s001.zip › ijms-3152692-supplementary.pdf]

Supplementary Table S1. Concentration of analytes by group (CM and non-CM).

Analyte	CM (n=11)		Non-CM (n=17)	
	Concentration (pg/ml)	Log_{10}	Concentration (pg/ml)	Log_{10}
Plasma				
IP10*	12775 (9275.45-84490)	4.11 (3.97-4.93)	2580.25 (1690-6166.75)	3.41 (3.23-3.79)
GRO	5954.5 (3619-8194)	3.77 (3.56-3.91)	12790 (7681.3-17716)	4.11 (3.89-4.25)
MIP-3α	93.74 (78.46-134.23)	1.97 (1.89-2.13)	63.16 (53.77-195.76)	1.8 (1.73-2.29)
IL-23	493.69 (291.9-1285.91)	2.69 (2.47-3.11)	720.49 (438.81-783.46)	2.86 (2.64-2.89)
MCP-1	660.86 (314.26-1382.5)	2.82 (2.5-3.14)	328.71 (159.41-1160.6)	2.52 (2.2-3.06)
Osteopontin	11309.25 (6700.5-23550.5)	4.05 (3.83-4.37)	5275.25 (2811-8677)	3.72 (3.45-3.94)
CSF				
IP10	10117.85 (4445.2-14195)	4.01 (3.65-4.15)	13035 (4911.6-163290)	4.12 (3.69-5.21)
GRO	204.71 (31.82-4700.66)	2.31 (1.5-3.67)	377.42 (105.71-932.92)	2.58 (2.02-2.97)
MIP-3α	33.41 (27.28-43.71)	1.52 (1.44-1.64)	37.95 (32.41-101.12)	1.58 (1.51-2)
IL-23	115.28 (35.56-129.07)	2.06 (1.55-2.11)	117 (48.45-125.71)	2.07 (1.69-2.1)
MCP-1	1547 (983.25-2504.13)	3.19 (2.99-3.4)	1768 (1530.75-3417.79)	3.25 (3.18-3.53)
Osteopontin*	11309.25 (6700.5-23550.5)	5.28 (5.12-5.9)	5275.25 (2811-8677)	4.49 (4.17-4.91)

* $p<0.05$
